# Supplementary material for: Sustainable Synthesis of Cadmium Sulfide, with Applicability in Photocatalysis, Hydrogen Production, and as an Antibacterial Agent, Using Two Mechanochemical Protocols
Source: Nanomaterials (Basel). 2022 Apr 7;12(8):1250. doi: 10.3390/nano12081250 (PMC9024533; doi:10.3390/nano12081250)
Supplement: Supplementary file 1 [file nanomaterials-12-01250-s001.zip › nanomaterials-1622935-supplementary.pdf]

nanomaterials

Supplementary Information

Sustainable Synthesis of Cadmium Sulfide, with Applicability in Photocatalysis, Hydrogen Production, and as an Antibacterial Agent, Using Two Mechanochemical Protocols

Zhandos Shalabayev [1,2,*], Matej Baláž [3], Natalya Khan [1], Yelmira Nurlan [1], Adrian Augustyniak [4,5], Nina Daneu [6], Batukhan Tatykayev [1], Erika Dutková [3], Gairat Burashev [1], Mariano Casas-Luna [7,8], Róbert Džunda [9], Radovan Bureš [9], Ladislav Čelko [7], Aleksandr Ilin [2] and Mukhambetkali Burkitbayev [1]

[1] General and Inorganic Chemistry Department, Al-Farabi Kazakh National University, Al-Farabi Ave. 71, Almaty 050040, Kazakhstan; natalya.khan@kaznu.edu.kz (N.K.); nurlan_yelmira1@kaznu.edu.kz (Y.N.); batukhan.tatykaev@kaznu.kz (B.T.); kairat.burashev@mail.ru (G.B.); mukhambetkali.burkitbayev@kaznu.edu.kz (M.B.)
[2] Scientific Center for Anti-Infectious Drugs, Al-Farabi Ave. 75B, Almaty 050060, Kazakhstan; ilin_ai@mail.ru
[3] Institute of Geotechnics, Slovak Academy of Sciences, Watsonova 45, 04001 Košice, Slovakia; balazm@saske.sk (M.B.); dutkova@saske.sk (E.D.)
[4] Chair of Building Materials and Construction Chemistry, Technische Universität Berlin, Gustav-Meyer-Allee 25, 13355 Berlin, Germany; adrian.augustyniak@zut.edu.pl
[5] Faculty of Chemical Technology and Engineering, West Pomeranian University of Technology in Szczecin, Piastów Ave. 42, 71-065 Szczecin, Poland
[6] Jožef Stefan Institute, Jamova Cesta 39, 01000 Ljubljana, Slovenia; nina.daneu@ijs.si
[7] Central European Institute of Technology, Brno University of Technology, Purkynova 123, 612 00 Brno, Czech Republic; casas@vutbr.cz (M.C.-L.); ladislav.celko@ceitec.vcutbr.cz (L.Č.)
[8] Department of Physics of Materials, Charles University, 121 16 Prague, Czech Republic
[9] Institute of Materials Research, Slovak Academy of Sciences, Watsonova 47, 04001 Košice, Slovakia; rdzunda@saske.sk (R.D.); rbures@saske.sk (R.B.)
* Correspondence: zhandos.shalabayev@kaznu.edu.kz; Tel.: +7-707-793-17-65

The supporting information document contains 8 pages, 6 figures, and 1 tables

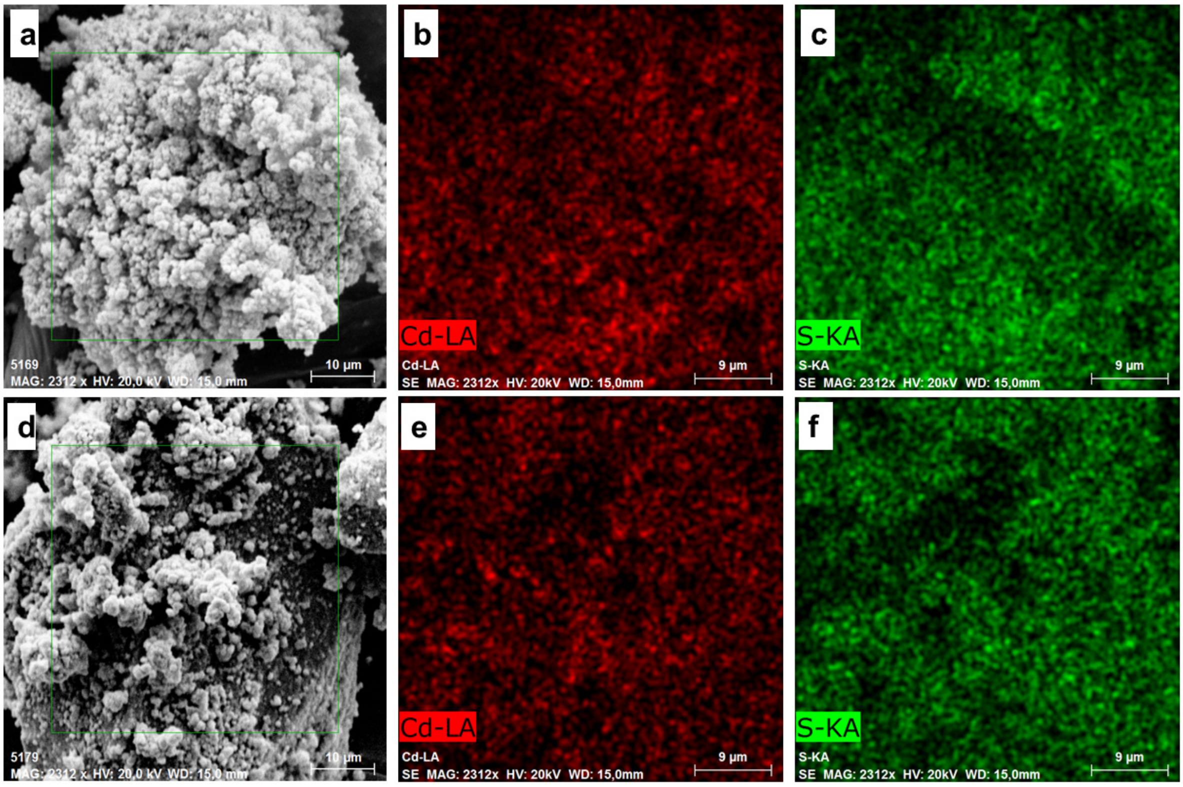

Figure S1. EDS elemental mapping of aCdS (top) and cCdS samples (bottom): (a,d) complex image showing the analyzed are in a green rectangle; (b,e) Cd mapping; (c,f) S mapping.

Table S1. Content of Cd and S in atomic % from distinct areas in each sample according to EDS analysis.

	Cd		S	
	**aCdS**	**cCdS**	**aCdS**	**cCdS**
**Area 1**	52.91	52.88	47.09	47.12
**Area 2**	53.42	54.15	46.58	45.85
**Area 3**	53.63		46.37	

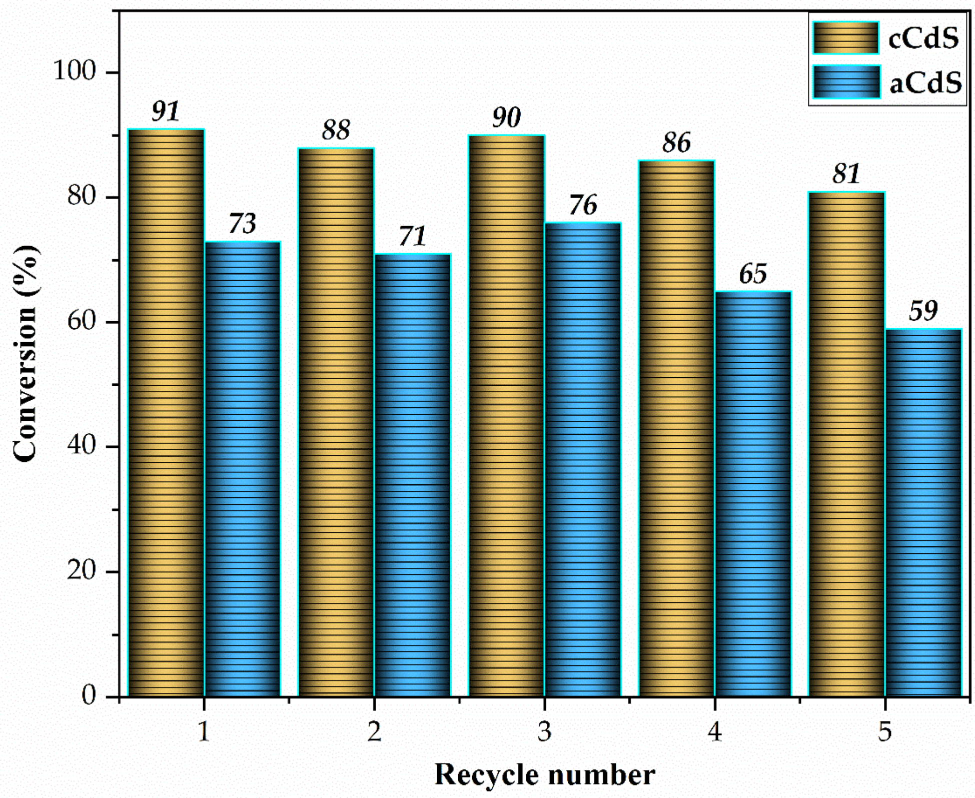

Figure S2. Results of the cyclic test of aCdS and cCdS samples for Orange II.

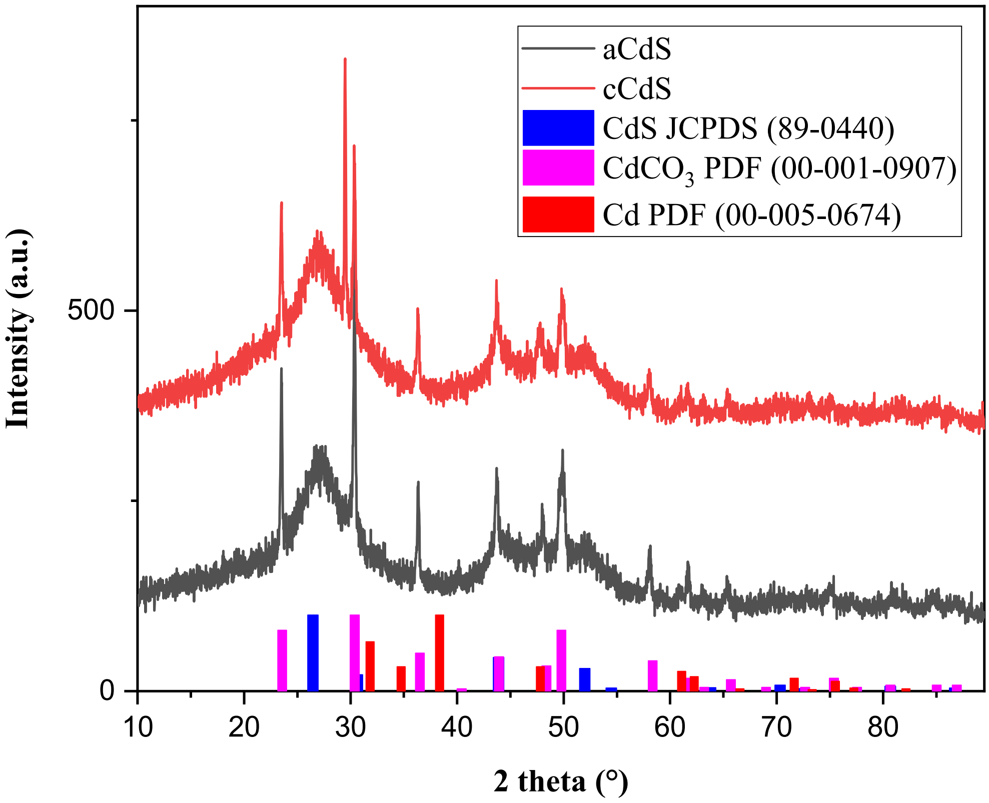

Figure S3. XRD pattern of remained cCdS and aCdS photocatalysts after performing stability tests of catalysts for Orange II dye.

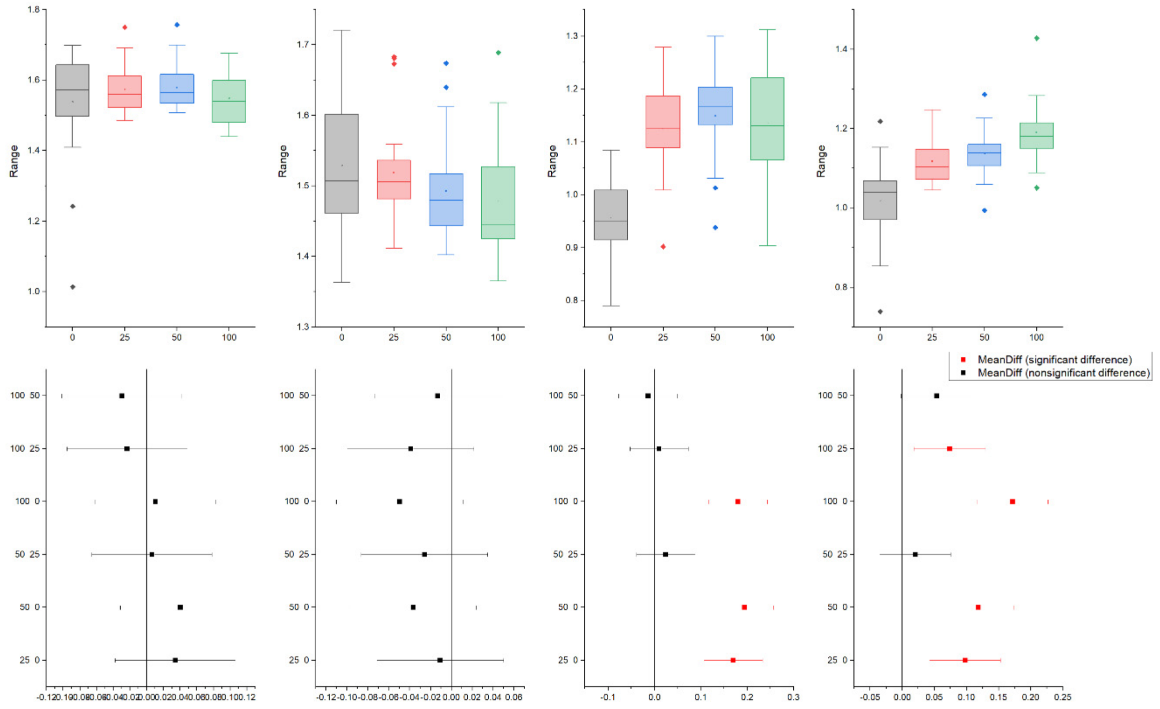

Figure S4. Statistical analysis of results obtained from optical density measurements after 24-hour incubation with nanomaterials or deionized water; in columns, starting from the left side: *S. aureus* with aCdS, *S. aureus* with cCdS, *E. coli* with aCdS, and *E. coli* with cCdS; bottom row presents results from Tukey's test.

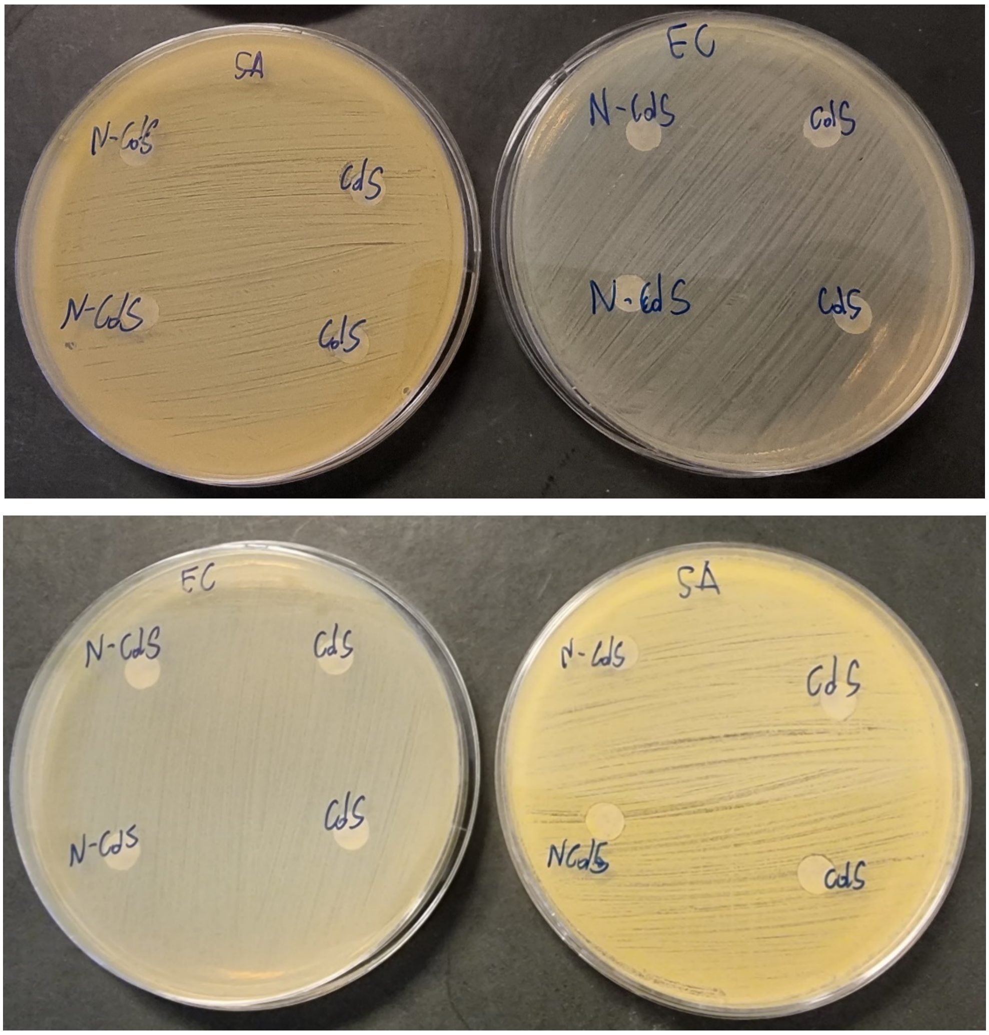

Figure S5. Disk assays for with the produced nanomaterials; EC – *E.coli*, SA – *S. Aureus*, N-CdS – cCdS, CdS – aCdS.

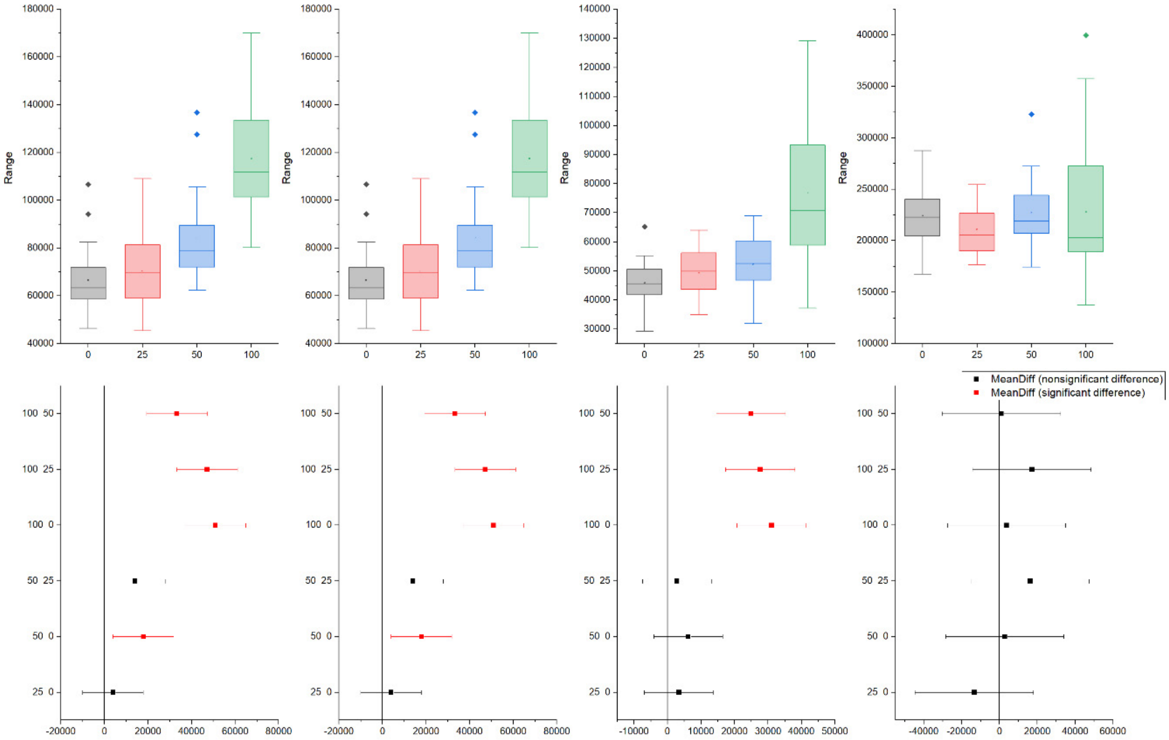

Figure S6. Statistical analysis of results obtained from resazurin assay (respiration) after 24-hour incubation with nanomaterials or deionized water; in columns, starting from the left side: *S. aureus* with cCdS, *S. aureus* with aCdS, *E. coli* with aCdS, and *E. coli* with cCdS; bottom row presents results from Tukey's test.
